# Pleiotropic association of *LIPC* variants with lipid and urinary 8-hydroxy deoxyguanosine levels in a Taiwanese population

**DOI:** 10.1186/s12944-019-1057-9

**Published:** 2019-05-10

**Authors:** Ming-Sheng Teng, Semon Wu, Lung-An Hsu, I-Shiang Tzeng, Hsin-Hua Chou, Cheng-Wen Su, Yu-Lin Ko

**Affiliations:** 1 Department of Research, Taipei Tzu Chi Hospital, Buddhist Tzu Chi Medical Foundation, New Taipei city, Taiwan; 2 Department of Life Science, Chinese Culture University, Taipei, Taiwan; 3 The First Cardiovascular Division, Department of Internal Medicine, Chang Gung Memorial Hospital and Chang Gung University College of Medicine, Taoyuan, Taiwan; 4 The Division of Cardiology, Department of Internal Medicine and Cardiovascular Center, Taipei Tzu Chi Hospital, Buddhist Tzu Chi Medical Foundation, New Taipei city, Taiwan; 5 School of Medicine, Tzu Chi University, Hualien, Taiwan

**Keywords:** Hepatic lipase, Single-nucleotide polymorphism, Mediation analysis, Suppression effect, Triglyceride level, High-density lipoprotein cholesterol level

## Abstract

**Background:**

Hepatic lipase (HL, encoded by *LIPC*) is a glycoprotein primarily synthesized and secreted by hepatocytes. Previous studies had demonstrated that HL is crucial for reverse cholesterol transport and affects the metabolism, composition, and level of several lipoproteins. In current study, we investigated the association of *LIPC* (Lipase C, Hepatic Type) variants with circulating and urinary biomarker levels by using subgroup and mediation analyses.

**Methods:**

A total of 572 participants from Taiwan were genotyped for three *LIPC* single nucleotide polymorphisms (SNPs) by using TaqMan assay. Fasting levels of glucose, lipid profile, inflammation markers, urine creatinine and 8-hydroxy deoxyguanosine (8-OHdG) were measured. The chi-square test, 2-sample *t* test and Analysis of variance (ANOVA) were used to examine differences among variables and genotype frequencies.

**Results:**

SNPs rs2043085 and rs1532085 were significantly associated with urinary 8-OHdG levels, whereas all three SNPs were more significantly associated with Triglycerides (TG) or HDL-cholesterol (HDL-C) levels after additional adjustment for HDL-C or TG levels, respectively. Subgroup analyses revealed that the association of the *LIPC* SNPs with the levels of serum TG, HDL-C, and urinary 8-OHdG were predominantly observed in the men but not in the women. Differential associations of the *LIPC* SNPs with various lipid levels were observed in participants with different adiposity statuses. Mediation analyses indicated that TG levels acted as a suppressor masking the association of the *LIPC* genotypes with HDL-C levels, particularly in the men (Sobel test, all *P* < 0.01).

**Conclusion:**

Our data revealed that interaction and suppression effects mediated the pleiotropic association of the *LIPC* variants. The effects of the *LIPC* SNPs depended on sex, adiposity status, and TG levels. Thus, our findings can provide a method for identifying high-risk populations of cardiovascular diseases for clinical diagnosis.

**Electronic supplementary material:**

The online version of this article (10.1186/s12944-019-1057-9) contains supplementary material, which is available to authorized users.

## Background

Hepatic lipase (HL, encoded by *LIPC*) is a glycoprotein primarily synthesized and secreted by hepatocytes [1]. It is a member of the triacylglycerol lipase family responsible for the hydrolysis of TGs and phospholipids [2]. It converts large, TG-rich HDL_2_ into small, dense HDL_3_ and is a negative regulator of plasma HDL cholesterol (HDL-C) levels [1]. In addition, it facilitates the uptake of chylomicron remnant-like particles by acting as a ligand for glycosaminoglycans on the surface of rat hepatocytes [1]. In humans, its deficiency increases the levels of large HDL_2_ particles, enriches HDL with TG, and causes hyperalphalipoproteinemia [1]. Taken together, HL is crucial for reverse cholesterol transport and affects the metabolism, composition, and levels of several lipoproteins [3, 4].

Numerous genome-wide association studies (GWAS) have found the associations between *LIPC* SNPs and lipid levels, most commonly that of HDL-C, in different ethnic populations [5–7]. However, inconsistent results have been reported regarding the association of the *LIPC* promoter SNP C-514 T/rs1800588 with TG and HDL-C levels [8, 9]. Interaction with sex, obesity, dietary intake, and physical activity further complicates these pleiotropic associations [10, 11]. Several groups have attempted to increase the statistical power by including a high number of participants into meta-analyses or phenome-wide association studies (PheWASs). However, except for HDL-C, the association of rs1800588 with various lipids has still not been consistently replicated [12–14]. The source of complexity may originate from a plethora of confounding factors, and other SNPs in linkage disequilibrium with rs1800588 may modify the association of rs1800588 with lipid traits.

HL activity has been linked to oxidative stress. For instance, the rs1800588-T allele is shown to reduce HL activity and increase the levels of malondialdehyde (MDA)-modified LDL [9], possibly via free radicals [15]. HL may affect the generation of reactive oxygen species (ROS) by modulating the activity of peroxisome proliferator-activated receptor δ (PPARδ), a key transcription factor known to counteract ROS production [16–18]. Because oxidized LDL are taken up by macrophage which enhanced foam cell formation, it is believed to increase the risk of systemic inflammation and atherosclerosis [19]. However, data from GWAS-related studies still have not provided concrete evidence for an association between rs1800588 and CAD [20, 21], and other *LIPC* SNPs facilitating the generation of oxidative stress signals may contribute to the risk of atherosclerosis and CAD.

Among all product of nucleic acids oxidation, 8-hydroxydeoxyguanosine (8-OHdG) is the most characterized. It is constantly excreted into the urine before and after a meal, and is stable in a freezer for up to a year [22]. High urinary 8-OHdG levels are positively associated with cancer, atherosclerosis, hypertension, chronic kidney diseases, and diabetes [23–27]. The ROS accumulates as these diseases progress, and the number of lesions escalates when more guanosine bases are damaged [28–30]. In contrast, urinary 8-OHdG is negatively associated with BMI [31]. Nevertheless, the close interplay between oxidative stress and disease progression suggests the usefulness of 8-OHdG as a marker for both conditions.

In addition, HL activity may play a role in the generation of inflammation. Constant exposure of the endothelium to products of lipolysis through the action of various vascular lipases, including HL, could trigger pro- or anti-inflammatory responses in and around the endothelial cells [32], depending on the activation or inactivation of downstream signal pathways. Mice with HL deficiency had fewer number of macrophage in the adipose tissue and ameliorate inflammation and macrophage proliferation by inactivating the LIGHT/lymphotoxin β-receptor pathway, and lessen the burden of atherosclerosis in mice with metabolic syndrome or insulin resistance [33]. Andres-Blasco et al. have shown that HL(−/−) mice fed with a high-fat, high-cholesterol diet had phenotypes consistent with increased inflammation including enhanced circulating monocyte chemotactic protein 1 (MCP1) levels and activation of stress-induced SAPK/JNK- and p38-MAPK pathways [34]. Therefore, examining the correlation between the *LIPC* SNPs and inflammatory marker levels in patients with cardiovascular disease can help us understand the role and contribution of HL to the development of this disease.

To understand the role of *LIPC* SNPs in determining lipid profiles and to elucidate the mechanisms underlying their effects, we included three SNPs in the 5′ region (rs2043085, rs1800588, and rs1532085) and analyzed their associations and mediation effects with various lipid traits in a Taiwanese population. We determined whether these SNPs contributed to oxidative stress or inflammation by using urinary 8-OHdG as an oxidative stress marker [25] and analyzed the correlation between its levels and these *LIPC* SNPs. Because we had previously determined the interactive effect of sex and obesity on the association of two *LIPC* promoter SNPs with lipid traits [8], we performed subgroup and mediation analyses in this extended study of *LIPC* variants.

## Methods

### Subjects

This study was approved by the Institutional Review Board of Taipei Tzu Chi Hospital, Buddhist Tzu Chi Medical Foundation. After obtaining informed consent, participants from the general population were consecutively recruited during routine cardiovascular health examinations. Only those without a known history of major systemic diseases or cardiovascular diseases were enrolled. Clinical history including hypertension, diabetes, habitual smoking, and drug use was recorded for all participants. Exclusion criteria included age < 18 years, pregnancy, cancer, and a history of myocardial infarction, stroke, or transient ischemic attack. In addition, participants with diabetes mellitus (defined as blood sugar level before meals ≥7.0 mmol/L or regular use of medications for diabetes mellitus) and macroalbuminuria (defined as a urinary albumin–creatinine ratio [ACR] > 300 mg/g) were excluded. Furthermore, to prevent the enrollment of patients with acute inflammatory disease, those with CRP levels > 10 mg/L [35] and using lipid-lowering medications were excluded. In total, 572 participants (296 men with a mean age of 45.0 ± 9.6 years and 276 women with a mean age of 46.5 ± 9.7 years) were enrolled. Participants who smoked at least one cigarette per day at the time of the survey were considered current smokers.

### Genomic DNA extraction and genotyping

Genomic DNA was extracted as previously described [36]. Three *LIPC* SNPs (rs2043085, rs1532085, and rs1800588) that have previously been found consistently associated with various metabolic phenotypes [7–11, 37] were selected according to the NCBI SNP database (http://www.ncbi.nlm.nih.gov/SNP). Genotyping was performed using TaqMan SNP Genotyping Assays from Applied Biosystems (ABI; Foster City, CA, USA). Basic characteristics and genotyping assays for the *LIPC* SNPs are listed in Additional file 1:Table S1. For quality control purposes, approximately 10% of the samples were re-genotyped blind, and identical results were obtained.

### Laboratory examinations

A total of 15 mL of venous blood and urine were collected the morning after an overnight fast. Serum and plasma samples were obtained through centrifugation at 3000×g for 15 min at 4 °C. Immediately after centrifugation, the serum and plasma samples were frozen and stored at − 80 °C prior to analyses. The circulating plasma levels of matrix metalloproteinase 2 (MMP2), soluble P-selectin (sP-selectin), and soluble TNF receptor II (sTNFRII) and the serum levels of matrix metalloproteinase 1 (MMP1) were measured using commercially available ELISA kits (R&D, Minneapolis, MN, USA). Circulating serum levels of CRP were determined using the particle-enhanced turbidimetric immunoassay technique (Siemens Healthcare Diagnostics Ltd., Camberley, UK). The increase in turbidity that accompanies aggregation is proportional to the CRP concentration. Other markers including serum amyloid A (SAA), homocysteine, soluble intercellular adhesion molecule-1 (sICAM1), soluble vascular cell adhesion molecule-1 (sVCAM1), soluble E-selectin (sE-selectin), matrix metalloproteinase 9 (MMP9), plasma monocyte chemotactic protein 1 (MCP1), urine creatinine, and 8-OHdG were measured using a sandwich ELISA developed in-house. For the in-house ELISA, we coated the microwell of microtiter plates with primary anti-target protein antibody and detected the captured target protein with biotin-conjugated detecting antibody. To speed up the assay, we added the sample and biotin-conjugated detecting antibody simultaneously into the antibody-coated well. The final signal was provided by Amdext streptavidin HRP conjugate. All in-house kits exhibited satisfactory correlation when compared with commercially available ELISA kits (Additional file 1:references). Overall, the intra- and inter-assay variability coefficients were within the range of 1.8 to 9.5%. EDTA was used to prepare plasma for the analysis of sP-selectin, MMP-2 and sTNFRII, while sodium citrate was used to prepare plasma for the analysis of fibrinogen.

Glucose levels were enzymatically determined using the hexokinase method, and total cholesterol (TC) and TG levels were measured through automatic enzymatic colorimetry. HDL-C levels were enzymatically measured after phosphotungsten/magnesium precipitation. LDL-C levels were calculated using the Friedewald formula; however, in patients with a TG level > 400 mg/dL, LDL-C levels were measured using commercial reagents with a standard protocol. Plasma fibrinogen levels were determined using the Clauss method adapted for a Sysmex CA1–1500 instrument (Kobe, Japan). Serum insulin levels were measured using an immunoradiometric assay (Bio-source, Nivelles, Belgium). The homeostasis model assessment of insulin resistance (HOMA-IR) index was calculated using the following formula: HOMA-IR = fasting serum insulin (μU/mL) × fasting plasma glucose (mmol/L)/22.5.

### Statistical analysis

The chi-square test was used to examine differences among categorical variables and to compare allele and genotype frequencies. The clinical characteristics of continuous variables were expressed as means ± standard deviations and tested using the 2-sample *t* test or ANOVA. A general linear model was applied to identify the major effect of each polymorphism on clinical phenotype variables, with age, sex, BMI, and smoking as confounding covariates. All biomarker levels were logarithmically transformed prior to statistical analyses to adhere to a normality assumption. The biomarkers had non-normal distributions after normality test were analyzed by using Kolmogorov-Smirnov test. The result was adjusted by false discovery rate (FDR) for multiple test correction and the regression coefficient with FDR *P* value < 0.05 was considered as significant. The analysis of deviation from the Hardy–Weinberg equilibrium (HWE) and the estimation of linkage disequilibrium between polymorphisms were performed using Golden Helix SVS Win32 7.3.1 (Golden Helix, Bozeman, MT). For multiple testing in the genetic association analysis, values of *P* ≤ 0.005 using a 2-sided test were considered statistically significant. Missing data were handled with listwise deletion. To investigate mediation effects exerted by TG levels on the association of the *LIPC* variants with HDL-C levels, a conceptual model was proposed, and four criteria were devised to evaluate the suppression effects of serum TG levels [38]. **Criterion** one: The independent variable (*LIPC* genotypes) must predict the mediator (TG levels). **Criterion** two: The mediator (TG levels) must predict the dependent variable (HDL-C) when adjusting for the independent variable. The mediation effect was calculated as the product of two regression coefficients from **criteria** one and two and reflected intermediate pathways from the independent variable to the dependent variable through the mediator. The regression coefficient denoting a relationship between the independent and dependent variables adjusted for the mediator was expressed as a direct effect. **Criterion** three: The independent variable must have a significant effect on the dependent variable and be expressed as the total effect. The total effect could also be obtained by summing the direct and mediation (indirect) effects. **Criterion** four: The mediation effect must be significant according to the procedure outlined by Sobel [39, 40]. In addition, a suppression effect may arise in a situation when the direct effect was greater than the total effect [41]. In this situation, the direct and indirect effects often had fairly similar magnitudes with opposite signs, which were entirely or partially offset, resulting in a zero or a nonzero but ultimately insignificant total effect [42]. The Sobel test [43] is the most commonly used method for examining the statistical significance of the mediation effect. Therefore, we used β coefficients and standard errors from the above model to perform the Sobel test. The Sobel test was performed using an interactive tool (http://www.quantpsy.org/sobel/sobel.htm) by which the null hypothesis (H_0_: αβ = 0) was tested. The test statistic S approximately distributed as Z was obtained by dividing the estimated mediation effect (αβ) with the standard error (δ). The reported *P* values were drawn from the unit normal distribution under the assumption of a Z value in which the mediated effect equaled zero in the population, with ±1.96 critical values containing the central 95% of the unit normal distribution.

## Results

### Baseline data

Demographic data, clinical biochemical data, lipid and inflammatory biomarker profiles, and urinary 8-OHdG levels of the participants, stratified by sex, are summarized in Table 1. A significantly higher percentage of the men were current smokers (*P* < 0.001). In addition, the men had significantly higher BMI (*P* < 0.001), HOMA-IR (*P* < 0.001), waist circumference (*P* < 0.001), waist/hip ratio (*P* < 0.001), and circulating levels of fasting plasma glucose (*P* < 0.001), serum insulin (*P* = 0.01), LDL-C (*P* = 0.036), TG (*P* < 0.001), sE-selectin (*P* < 0.001), sP-selectin (*P* = 0.005), MMP-9 (*P* = 0.003), sTNFRII (*P* = 0.018), and MCP-1 (< 0.001) than did the women. By contrast, circulating HDL-C (*P* < 0.001), MMP-2 levels (*P* = 0.018) and urine 8-OHdG levels (*P* < 0.001) were lower in the men than in the women. For the studied polymorphisms, no significant difference in genotype frequencies between the men and women and no significant deviation from the HWE were observed (Additional file 1: Table S1).

**Table 1:** Clinical and biochemical characteristics of the study population

	Total	Men	Women	*P* value
Number	572	296	276	
Age (years)	45.7 ± 9.7	45.0 ± 9.6	46.5 ± 9.7	0.076
BMI (kg/m^2^)	24.2 ± 3.4	24.9 ± 3.0	23.5 ± 3.6	< 0.001
Fasting plasma glucose (mg/dL)	92.0 (88.0, 98.0)	94.0 (89.0, 100.0)	90.5 (86.0, 96.0)	< 0.001
Fasting serum insulin (μU/mL)	7.8 (6.1, 10.6)	8.3 (6.4, 11.4)	7.5 (5.9, 10.3)	0.01
HOMAIR	1.8 (1.4, 2.5)	2.0 (1.5, 2.6)	1.7 (1.3, 2.4)	< 0.001
Waist circumference (cm)	84.8 ± 9.4	87.7 ± 7.4	81.7 ± 10.3	< 0.001
Waist hip ratio	0.9 ± 0.1	0.9 ± 0.0	0.8 ± 0.1	< 0.001
Current smokers (%)	19.4	33.8	4.0	< 0.001
Hypertension (%)	8.9	8.0	9.8	0.309
Diabetes mellitus (%)	3.3	4.1	2.5	0.312
Total cholesterol (mg/dL)	199.3 ± 35.9	201.6 ± 35.8	196.8 ± 36.0	0.089
LDL-C (mg/dL)	116.5 ± 32.9	119.4 ± 33.7	113.5 ± 31.8	0.036
HDL-C (mg/dL)	54.0 (45.0, 65.0)	48.0 (42.0, 55.0)	61.0 (51.3, 71.0)	< 0.001
TG (mg/dL)	114.5 (76.0, 164.8)	135.0 (92.0, 203.8)	91.0 (67.0, 134.8)	< 0.001
CRP (mg/L)	0.6 (0.3, 1.2)	0.6 (0.3, 1.2)	0.5 (0.2, 1.2)	0.098
Fibrinogen (mg/dL)	260.3 ± 67.3	256.9 ± 68.8	263.8 ± 65.6	0.187
sE-selectin(ng/L)	49.9 (35.6, 64.3)	57.0 (41.6, 73.5)	42.5 (31.1, 54.7)	< 0.001
sP-selectin(ng/mL)	95.0 (65.9, 169.7)	103.9 (70.0, 187.8)	86.7 (60.0, 151.3)	0.005
SAA (μmol/L)	3.6 (1.7, 6.0)	3.3 (1.6, 6.0)	3.6 (1.7, 6.1)	0.399
sICAM1 (ng/L)	228.0 (179.9, 275.1)	232.0 (182.0, 283.6)	225.1 (175.8, 271.6)	0.776
sVCAM1(ng/L)	477.0 (406.0, 545.0)	479.0 (399.0, 555.0)	476.0 (415.0, 540.0)	0.81
MMP-1 (pg/mL)	188.3 (102.1, 398.8)	184.8 (110.8, 404.4)	189.0 (96.2, 398.4)	0.601
MMP-2 (ng/mL)	122.2 (103.1, 140.4)	118.9 (98.7, 137.5)	124.9 (106.9, 146.1)	0.018
MMP-9 (mg/L)	109.9 (73.8, 167.3)	120.5 (83.3, 184.6)	99.4 (66.1, 150.2)	0.003
MCP-1 (pg/mL)	59.8 (43.6, 83.5)	62.6 (46.1, 86.7)	56.2 (40.9, 80.8)	0.005
sTNFRII (pg/mL)	3095.8 (2635.9, 3708.9)	3120.2 (2742.4, 3726.3)	3066.8 (2528.2, 3700.6)	0.018
8-OHdG/creatinine (ng/mg)	32.6 (24.3, 44.2)	30.4 (22.5, 40.0)	35.2 (26.8, 48.4)	< 0.001
LIPC-rs2043085				
CC	159 (28.3%)	73 (25.2%)	86 (31.6%)	0.198
TC	284 (50.5%)	150 (51.7%)	134 (49.3%)	
TT	119 (21.2%)	67 (23.1%)	52 (19.1%)	
LIPC-rs1532085				
GG	162 (28.8%)	74 (25.5%)	88 (32.2%)	0.200
GA	287 (51.0%)	153 (52.8%)	134 (49.1%)	
AA	114 (20.2%)	63 (21.7%)	51 (18.7%)	
LIPC-rs1800588				
CC	228 (40.5%)	125 (43.1%)	103 (37.7%)	0.659
TC	261 (46.4%)	128 (44.1%)	133 (48.7%)	
TT	74 (13.1%)	37 (12.8%)	37 (13.6%)	

*BMI* body mass index, *HOMA-IR* homeostasis model assessment of insulin resistance, *HDL-C* high-density lipoprotein cholesterol, *LDL-C* low-density lipoprotein cholesterol, *TG* Triglycerides, *CRP* C-reactive protein, *sE-selectin* soluble E-selectin, *sP-selectin* soluble P-selectin, *SAA* serum amyloid A, *sICAM1* soluble intercellular adhesive molecule 1, *sVCAM1* soluble vascular cell adhesive molecule 1, *MMP1* matrix metalloproteinase 1, *MMP2* matrix metalloproteinase 2, *MMP9* matrix metalloproteinase 9, *MCP-1* Monocyte chemotactic protein-1, *sTNFRII* soluble tumor necrosis factor-alpha receptor 2, *8-OHdG* 8-hydroxy deoxyguanosine. Continuous variables are presented as mean ± SD. Fasting plasma glucose, fasting serum insulin, HOMAIR, HDL-C, TG, CRP, sE-selectin, sP-selectin, SAA, sICAM1, sVCAM1, MMP1, MMP2, MMP9, MCP1, sTNFRII and 8-OHdG values were logarithmically transformed before statistical testing to meet the assumption of normal distributions; however, the untransformed data are shown as median (range)

All variables had non-normal distributions after Normality test, including fasting plasma glucose, fasting serum insulin, HOMAIR, sE-selectin, sP-selectin, SAA, sICAM1, MMP1, MMP2, MMP9, MCP1 and sTNFRII were analyzed by using Kolmogorov-Smirnov test

### Relationship between the LIPC SNPs and lipid variables

To determine the effects of the *LIPC* genotypes on lipid levels, we created an additive model by using four lipid traits as variables of interest (Table 2). After adjustment for age, sex, smoking status, and BMI, compared with the participants carrying the major alleles of the studied SNPs, those carrying the minor alleles exhibited a trend of higher TG levels (*P* = 0.007, *P* = 0.006, and *P* = 0.009 for rs2043085, rs1532085, and rs1800588, respectively). The association became more significant after further adjustment for HDL-C levels and FDR corrections (*P* = 2.7 × 10^− 4^, *P* = 2.7 × 10^− 4^, and *P* = 4.5 × 10^− 4^, respectively). Although unadjusted *P* values were bordering the cutoff threshold, the association of the studied SNPs with HDL-C levels became much more significant after further adjustment for serum TG levels and FDR corrections (*P* = 5.4 × 10^− 4^, *P* = 4.5 × 10^− 4^, and *P* = 0.002 for rs2043085, rs1532085, and rs1800588, respectively). In addition, a significant association of the rs1800588 genotype with TC levels was observed (*P* = 0.003).

**Table 2:** Associations between the *LIPC* SNPs and lipid profiles and urinary 8OHdG levels

Genotypes	MM	Mm	mm	*P1* (adjusted *P*)	*P2* (adjusted *P*)
rs2043085, number	159	284	119		
Total cholesterol (mg/dL)	198.8 ± 39.7	198.3 ± 33.6	201.6 ± 35.8	0.435	
LDL-C (mg/dL)	120.2 ± 37.9	114.8 ± 30.2	115.2 ± 31.4	0.248	
HDL-C (mg/dL)	53.0 (44.0, 64.0)	53.0 (44.0, 66.0)	54.0 (45.0, 65.0)	0.039	^*a*^ 3 × 10^− 4^ (5.4 × 10^− 4^)
TG (mg/dL)	102.0 (71.0, 151.0)	111.0 (75.3, 161.8)	129.0 (90.0, 186.0)	0.007 (0.021)	^b^6 × 10^−5^ (2.7 × 10^− 4^)
8-OHdG (ng/mg)	32.9 (21.8, 44.1)	31.6 (24.7, 43.0)	35.9 (27.4, 47.6)	0.001 (0.015)	^*a*^4 × 10^− 4^ (6.0 × 10^− 4^)
rs1532085, number	162	287	114		
Total cholesterol (mg/dL)	198.1 ± 40.0	198.6 ± 33.3	201.7 ± 35.9	0.309	
LDL-C (mg/dL)	119.5 ± 38.2	115.2 ± 30.0	114.9 ± 31.3	0.348	
HDL-C (mg/dL)	53.0 (44.0, 63.3)	53.0 (44.0, 66.0)	54.0 (45.0, 65.3)	0.035	^*a*^2 × 10^−4^ (4.5 × 10^− 4^)
TG (mg/dL)	101.5 (70.8, 151.5)	112.0 (76.0, 161.0)	129.0 (90.0, 196.3)	0.006 (0.021)	^b^5 × 10^−5^ (2.7 × 10^− 4^)
8-OHdG (ng/mg)	32.6 (22.4, 44.1)	32.1 (24.8, 43.5)	35.8 (26.9, 46.6)	0.002 (0.015)	^*a*^0.001 (0.001)
rs1800588, number	228	261	74		
Total cholesterol (mg/dL)	195.2 ± 35.3	200.3 ± 36.1	206.7 ± 35.7	0.003 (0.015)	
LDL-C (mg/dL)	115.2 ± 33.6	116.6 ± 32.7	118.9 ± 31.1	0.196	
HDL-C (mg/dL)	52.0 (44.3, 64.0)	54.0 (44.0, 65.0)	56.5 (46.8, 69.0)	0.089	^*a*^0.002 (0.002)
TG (mg/dL)	107.0 (72.3, 161.8)	116.0 (76.0, 164.5)	118.5 (79.0, 183.5)	0.009 (0.023)	^b^2 × 10^−4^ (4.5 × 10^− 4^)
8-OHdG (ng/mg)	32.4 (24.3, 43.3)	33.7 (25.5, 45.1)	29.7 (22.3, 39.4)	0.155	^*a*^0.177

Abbreviations as in Table 1

*P1*: adjusted for age, sex, BMI, and current smoke; *P2*: *P* values of associations between SNPs and HDL-C, 8-OHdG or TG levels after adjustment for age, sex, BMI, current smoke and TG (^*a*^) or HDL-C (^b^) levels

MM: homozygosity of major allele, Mm: heterozygosity, mm: homozygosity of minor allele

Adjusted *P* values were shown with False Discovery Rate correction of *P1 and P2* values, and only significant *P* values were demonstrated

### Associations of the LIPC SNPs with inflammatory biomarkers and urinary 8-OHdG levels

To determine whether the *LIPC* genotypes influenced inflammatory marker levels, we analyzed the levels of the following 12 inflammatory markers: CRP, fibrinogen, SAA, sICAM1, sVCAM1, sE-selectin, sP-selectin, MMP1, MMP-2, MMP-9, MCP1, and sTNFRII. No significant differences were observed in all the analyzed inflammatory marker levels among the *LIPC* genotypes (Additional file 1: Table S2–S4). Table 2 lists the variations in urinary 8-OHdG levels across the *LIPC* genotypes. After the additive model was adjusted for age, sex, smoking status, and BMI, rs2043085 and rs1532085 genotypes were significantly associated with urinary 8-OHdG levels (*P* = 0.001 and *P* = 0.002, respectively). By contrast, no significant association of the rs1800588 genotype with urinary 8-OHdG levels was observed.

### Interaction of the LIPC SNPs, lipid variables, and urinary 8-OHdG levels with sex

As depicted in Table 3, in the additive model, the men carrying the minor alleles of the studied SNPs had significantly higher TG levels than did those carrying the major alleles (*P* = 0.005, *P* = 0.005, and *P* = 0.004 for rs2043085, rs1532085, and rs1800588, respectively), and the associations became more significant after further adjustment for HDL-C levels and FDR corrections (*P* = 6 × 10^− 4^, *P* = 4.5 × 10^− 4^, and *P* = 3.6 × 10^− 4^, respectively). By contrast, HDL-C levels were significantly associated with the *LIPC* genotypes in the men only after adjustment for serum TG levels and FDR corrections (*P* = 0.006, *P* = 0.005, and *P* = 0.002 for rs2043085, rs1532085, and rs1800588, respectively). In addition, after adjusting age, sex, smoking status, and BMI for the men, the rs2043085 genotype was significantly associated with urinary 8-OHdG levels (*P* = 0.001), whereas the rs1532085 genotype demonstrated a trend of the association (*P* = 0.007). Furthermore, a significant association of the rs1800588 genotype with TC levels was observed only in the men (*P* = 0.003). None of the above associations was observed in the women after FDR correction. These results suggest that the associations of the *LIPC* SNPs with the lipid variables and urinary 8-OHdG levels were dependent on sex.

**Table 3:** Subgroup analysis for the associations between the *LIPC* SNPs and lipid profiles and urinary 8OHdG levels according to sex

Sex	Male					Female				
Genotypes (N)	MM	Mm	mm	*P1* *(*adjusted *P)*	*P2* *(*adjusted *P)*	MM	Mm	mm	*P1* *(*adjusted *P)*	*P2* *(*adjusted *P)*
rs2043085 number	73	150	67			86	134	52		
Total cholesterol (mg/dL)	199.9 ± 42.2	199.6 ± 33.8	205.2 ± 32.4	0.300		197.9 ± 37.8	196.9 ± 33.4	196.9 ± 39.5	0.781	
LDL-C (mg/dL)	125.4 ± 40.3	115.8 ± 31.4	118.5 ± 29.8	0.339		115.8 ± 35.5	113.6 ± 28.7	110.9 ± 33.2	0.299	
HDL-C (mg/dL)	47.0 (40.5, 53.5)	49.0 (42.8, 55.3)	50.0 (43.0, 56.0)	0.194	^*a*^0.005 (0.006)	59.5 (51.0, 70.3)	62.0 (52.0, 71.0)	62.0 (52.3, 70.8)	0.081	^*a*^0.028
TG (mg/dL)	126.0 (78.5, 162.5)	135.5 (93.0, 222.3)	150.0 (105.0, 218.0)	0.005 (0.015)	^b^2 × 10^−4^ (6 × 10^− 4^)	90.0 (64.0, 135.0)	86.5 (65.8, 134.3)	105.0 (75.8, 142.0)	0.629	^b^0.151
8OHdG (ng/mg)	29.6 (20.6, 37.3)	30.0 (22.5, 38.8)	35.1 (24.3, 45.1)	0.001 (0.015)	^*a*^0.001 (0.002)	35.2 (23.3, 48.8)	34.5 (26.5, 45.9)	37.7 (28.2, 53.8)	0.088	^*a*^0.088
rs1532085 number	74	153	63			88	134	51		
Total cholesterol (mg/dL)	198.8 ± 42.6	199.9 ± 33.4	206.0 ± 32.2	0.173		197.4 ± 38.0	197.1 ± 33.2	196.3 ± 39.7	0.762	
LDL-C (mg/dL)	124.1 ± 40.9	116.3 ± 31.2	118.7 ± 29.4	0.516		115.5 ± 35.6	113.8 ± 28.5	110.3 ± 33.2	0.278	
HDL-C (mg/dL)	46.5 (40.0, 53.3)	49.0 (43.0, 55.8)	50.0 (41.0, 56.0)	0.183	^*a*^0.004 (0.005)	60.0 (51.0, 69.8)	62.0 (51.0, 71.0)	64.0 (53.0, 71.0)	0.079	^*a*^0.025
TG (mg/dL)	128.0 (78.8, 165.0)	131.0 (93.0, 220.0)	152.0 (105.0, 218.0)	0.005 (0.015)	^b^1.5 × 10^−4^ (4.5 × 10^− 4^)	90.0 (64.0, 133.8)	86.5 (66.8, 136.0)	104.0 (75.0, 142.0)	0.605	^b^0.140
8OHdG (ng/mg)	29.9 (20.2, 38.0)	30.0 (22.8, 38.9)	34.4 (23.5, 44.8)	0.007 (0.018)	^*a*^0.004 (0.005)	34.9 (23.2, 48.3)	34.8 (26.7, 47.3)	37.5 (28.1, 50.8)	0.085	^*a*^0.086
rs1800588 number	125	128	37			103	133	37		
Total cholesterol (mg/dL)	195.8 ± 36.6	202.4 ± 34.5	213.3 ± 34.3	0.003 (0.015)		194.5 ± 33.8	198.3 ± 37.5	200.0 ± 36.3	0.244	
LDL-C (mg/dL)	118.0 ± 35.9	118.6 ± 32.2	122.5 ± 31.3	0.336		111.9 ± 30.4	114.7 ± 33.1	115.3 ± 31.0	0.402	
HDL-C (mg/dL)	47.0 (42.5, 53.0)	48.0 (41.0, 57.0)	53.0 (43.0, 59.0)	0.100	^*a*^0.001 (0.002)	62.0 (50.0, 70.0)	61.0 (52.0, 70.0)	63.0 (54.5, 73.5)	0.423	^*a*^0.27
TG (mg/dL)	120.0 (89.0, 173.5)	145.5 (97.5, 197.8)	152.0 (106.0, 234.5)	0.004 (0.015)	^b^4.9 × 10^−5^ (3.6 × 10^− 4^)	90.0 (64.0, 140.0)	95.0 (67.5, 134.0)	87.0 (65.5, 135.0)	0.682	^b^0.387
8OHdG (ng/mg)	31.6 (24.4, 39.0)	29.9 (21.1, 42.8)	25.6 (21.3, 37.8)	0.098	^*a*^0.134	34.4 (24.1, 49.2)	37.3 (28.7, 49.6)	30.7 (23.3, 43.4)	0.698	^*a*^0.699

*P1*: adjusted for age, sex, BMI, current smoke; *P2*: *P* values of associations between SNPs and HDL-C, 8-OHdG or TG after adjusted for age, sex, BMI, current smoke and TG (^*a*^) or HDL-C (^b^)

MM: homozygosity of major allele, Mm: heterozygosity, mm: homozygosity of minor allele

Adjusted *P* values were shown with False Discovery Rate correction of *P1 and P2* values, and only significant *P* values were demonstrated

### Interaction of the LIPC SNPs, lipid variables, and urinary 8-OHdG levels with obesity

We stratified the participants according to their adiposity statuses. As illustrated in Table 4, in the additive model, only the nonobese carriers of the minor alleles of rs2043085 and rs1532085 had significantly higher TG levels than did those carrying the major alleles (*P* = 0.002 and *P* = 0.002, respectively). By contrast, only the obese carriers of the minor alleles of rs2043085 and rs1532085 had at least a trend of significantly higher HDL-C and urinary 8-OHdG levels than did those carrying the major alleles (*P* = 0.001 and *P* = 0.001 for HDL-C levels and *P* = 0.002 and *P* = 0.017 for urinary 8-OHdG levels, respectively). Furthermore, a significant association of the rs1800588 genotype with TC and LDL-C levels was observed only in the obese participants (*P* = 2 × 10^− 4^ and *P* = 0.003, respectively). These results suggest that differential associations of the *LIPC* SNPs with the lipid variables and urinary 8-OHdG levels were dependent on adiposity status.

**Table 4:** Subgroup analysis for the associations between the *LIPC* SNPs and lipid profiles and urinary 8OHdG levels according to obesity

Obesity	Obese					Non-obese				
Genotypes	MM	Mm	mm	*P1* *(*adjusted *P)*	*P2* *(*adjusted *P)*	MM	Mm	mm	*P1 (*adjusted *P)*	*P2* *(*adjusted *P)*
rs2043085 number	41	123	50			118	161	69		
Total cholesterol (mg/dL)	204.0 ± 41.3	201.0 ± 34.0	205.6 ± 35.4	0.6		197.0 ± 39.2	196.3 ± 33.2	198.7 ± 36.0	0.538	
LDL-C (mg/dL)	127.0 ± 39.8	116.3 ± 29.6	121.8 ± 31.6	0.754		117.8 ± 37.2	113.6 ± 30.6	110.3 ± 30.6	0.261	
HDL-C (mg/dL)	46.0 (40.5, 54.5)	49.0 (41.0, 59.0)	55.0 (47.0, 60.3)	0.001 (0.005)	^*a*^4 × 10^−4^ (0.002)	56.5 (46.8, 66.0)	58.0 (47.5, 70.0)	52.0 (44.0, 68.0)	0.717	^*a*^0.028 (0.032)
TG (mg/dL)	138.0 (103.0, 173.5)	141.0 (97.0, 223.0)	133.0 (91.8, 174.3)	0.995	^b^0.181	89.0 (64.8, 145.8)	92.0 (66.5, 128.5)	123.0 (88.0, 211.5)	0.002 (0.015)	^b^1 × 10^−4^ (5 × 10^−4^)
8OHdG (ng/mg)	30.0 (19.1, 37.7)	31.1 (23.8, 40.2)	36.3 (25.6, 55.1)	0.002 (0.008)	^*a*^0.003 (0.009)	33.3 (23.1, 44.7)	32.7 (24.7, 44.0)	35.9 (27.8, 45.9)	0.035	^*a*^0.026 (0.032)
rs1532085 number	42	124	48			120	163	66		
Total cholesterol (mg/dL)	202.1 ± 42.3	201.6 ± 33.7	205.8 ± 35.3	0.398		196.6 ± 39.3	196.3 ± 33.0	198.7 ± 36.3	0.515	
LDL-C (mg/dL)	124.9 ± 41.0	117.2 ± 29.3	121.7 ± 31.4	0.974		117.6 ± 37.2	113.7 ± 30.5	110.0 ± 30.6	0.270	
HDL-C (mg/dL)	46.0 (40.0, 54.3)	49.5 (42.0, 59.0)	55.0 (47.0, 60.8)	0.001 (0.005)	^*a*^3 × 10^−4^ (0.002)	57.0 (47.0, 65.8)	57.0 (47.0, 70.0)	52.5 (43.8, 68.0)	0.708	^*a*^0.028 (0.032)
TG (mg/dL)	141.0 (105.5, 176.8)	140.5 (96.3, 220.5)	133.0 (94.0, 176.8)	0.968	^b^0.160	89.0 (64.3, 143.3)	93.0 (67.0, 135.0)	118.5 (85.8, 210.3)	0.002 (0.015)	^b^1.4 × 10^−4^ (5 × 10^−4^)
8OHdG (ng/mg)	30.7 (18.8, 39.0)	31.1 (24.0, 40.8)	35.8 (24.1, 47.3)	0.017 (0.043)	^*a*^0.017 (0.031)	33.1 (23.1, 44.5)	33.0 (25.1, 44.3)	35.5 (27.5, 46.6)	0.030	^*a*^0.021 (0.032)
rs1800588 number	91	97	26			137	164	48		
Total cholesterol (mg/dL)	193.5 ± 36.6	206.4 ± 32.5	220.4 ± 36.4	2 × 10^−4^ (0.003)		196.3 ± 34.5	196.7 ± 37.7	199.3 ± 33.4	0.484	
LDL-C (mg/dL)	113.2 ± 33.7	122.4 ± 30.3	132.0 ± 31.0	0.003 (0.009)		116.6 ± 33.5	113.2 ± 33.6	111.8 ± 29.2	0.538	
HDL-C (mg/dL)	50.0 (42.0, 56.0)	51.0 (43.0, 61.0)	53.5 (43.0, 58.5)	0.367	^*a*^0.060	55.0 (46.0, 68.0)	57.0 (47.0, 67.8)	62.0 (50.3, 72.5)	0.136	^*a*^0.012 (0.032)
TG (mg/dL)	132.0 (86.0, 174.0)	140.0 (105.5, 186.5)	141.5 (119.3, 216.8)	0.028	^b^0.006 (0.014)	93.0 (67.0, 134.5)	98.5 (68.5, 154.5)	90.5 (67.3, 167.3)	0.156	^b^0.014 (0.032)
8OHdG (ng/mg)	32.0 (23.3, 42.7)	31.5 (22.2, 43.3)	30.1 (19.1, 38.3)	0.13	^*a*^0.144	33.0 (24.9, 43.9)	34.7 (26.2, 46.0)	29.0 (23.1, 41.7)	0.59	^*a*^0.619

*P1*: adjusted for age, sex, BMI, current smoke; *P2*: *P* values of associations between SNPs and HDL-C, 8-OHdG or TG levels after adjusted for age, sex, BMI, current smoke and TG (^*a*^) or HDL-C (^b^)

MM: homozygosity of major allele, Mm: heterozygosity, mm: homozygosity of minor allele

Adjusted *P* values were shown with False Discovery Rate correction of *P1 and P2* values, and only significant *P* values were demonstrated

### Serum TG levels suppressed the sex-dependent association of the LIPC SNPs with HDL-C levels

**Fig. 1:**
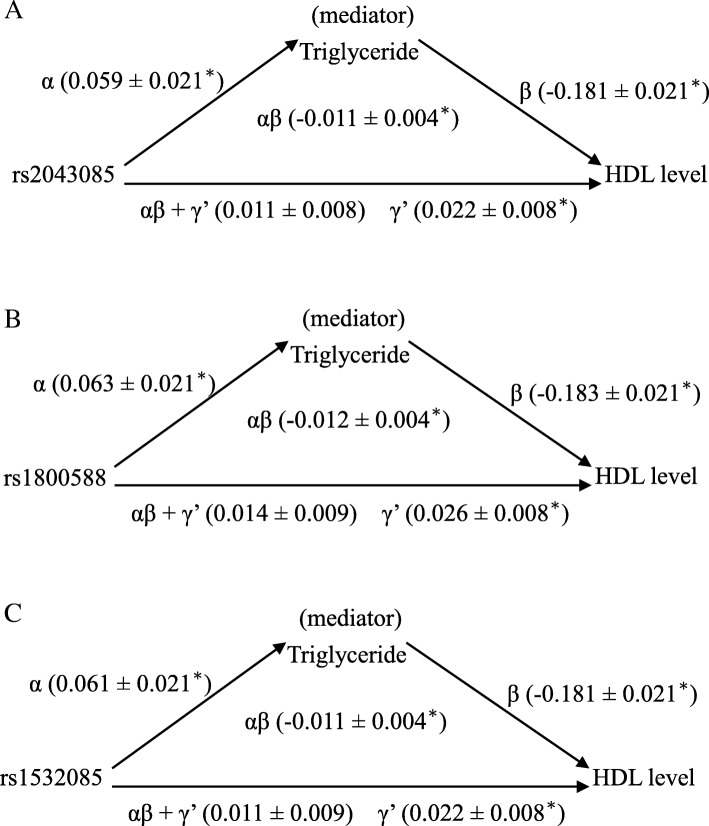
A three-variable mediation model in the males with TG levels as a mediator for the associations between the *LIPC* genotypes and HDL-C levels**.** Linear regression models were used to assess the following path associations, exemplified in (**a**). Relationships between (α) the *LIPC* genotype and TG levels, (β) the TG and HDL-C levels, (αβ + γ’) the *LIPC* genotypes and HDL-C levels, and (γ’) the *LIPC* genotypes and HDL-C levels after adjustment for the TG levels were shown. Each estimate along the path represented the unstandardized β coefficient from the regression model. The results indicated that the *LIPC* genotypes exhibited a stronger association with the HDL-C levels after adjustment for the TG levels. The direct effects (γ’) of the *LIPC* genotypes on the HDL-C levels (0.022) were greater than the total effects (αβ + γ’) (0.011), which had opposite signs to those of the mediation effects (αβ) (− 0.011), and suggested significant mediation (suppression) by the TG levels. All models were adjusted for age, BMI, and current smoking status. ^*^P < 0.01. In addition, the other analyses (**b** and **c**) exhibited similar suppression effects. Suppression triangles obtained from the male subjects

Four criteria were applied to study the mediation and suppression effects in the men carrying different *LIPC* genotypes (Table 5). In brief, the *LIPC* genotypes were significantly associated with TG levels (criterion 1), which in turn had significant negatively effects on HDL-C levels (criterion 2). The total effect of the *LIPC* genotypes on HDL-C levels was 0.011, 0.014, and 0.011 for rs2043085, rs1800588, and rs1532085, respectively, with insignificant *P* values (criterion 3). The Sobel test for the mediation of the results of the corresponding HDL-C levels revealed that z was − 2.8, − 3, and − 2.8, respectively (all *P* < 0.01, criterion 4). Moreover, the direct effects (γ’) of the *LIPC* genotypes on HDL-C levels were higher than their total effects (αβ + γ’), which had similar magnitudes as those of the mediation effects but with opposite signs (αβ), demonstrating a suppression effect in this model (Fig. 1).

**Table 5:** Mediation tests of the TG levels on the associations between the *LIPC* genotypes and HDL-C levels in male participants

		rs2043085	rs1800588	rs1532085
Criterion 1	α regression coefficient Standard error *P* value			
		0.059 0.021 0.005	0.063 0.021 0.004	0.061 0.021 0.005
Criterion 2	β regression coefficient Standard error ^#^*P* value			
		−0.181 0.021 7.2 × 10^−16^	−0.183 0.021 2.9 × 10^−16^	− 0.181 0.021 6.5 × 10^− 16^
	γ’ regression coefficient Standard error *P value			
		0.022 0.008 0.005	0.026 0.008 0.001	0.022 0.008 0.004
Criterion 3	αβ + γ’ regression coefficient Standard error *P* value			
		0.011 0.008 0.194	0.014 0.009 0.100	0.011 0.009 0.183
Criterion 4	αβ regression coefficient Standard error *P* value (Sobel test)			
		−0.011 0.004 0.008	−0.012 0.004 0.005	− 0.011 0.004 0.006

*LIPC* genotypes were analyzed in dominant models,

α: unstandardized coefficient for the association between *LIPC* genotypes and TG levels

β: unstandardized coefficient for the association between TG and HDL-C levels (when adjusting for *LIPC* genotypes)

Direct effect = γ’, Total effect = αβ + γ’, Mediation (indirect) effect = αβ

*P*: adjusted for age, sex, BMI, current smoke; *: indicated the *P* value of association between SNPs and HDL-C after adjusted for age, sex, BMI, current smoke and TG; ^#^: indicated the *P* value of association between TG and HDL-C after adjusted for age, sex, BMI, current smoke and SNPs

## Discussion

This study investigated the association of *LIPC* SNPs with various circulating lipid and biomarker levels. Our data revealed a significant association of three *LIPC* SNPs with TC, HDL-C, and TG levels, with the interaction of sex and obesity; this finding was consistent with that of our previous study [8]. In addition, we observed that TG levels exerted a suppression effect on the association of *LIPC* SNPs with HDL-C levels. Furthermore, the association of two *LIPC* SNPs with urinary 8-OHdG levels, a marker of systemic oxidative stress, was observed. The involvement of both pro- and anti-atherosclerotic risk factors observed in this study may have meaningful implications for preventive medicine. Our findings might facilitate the identification of high-risk populations in preventive medicine of cardiovascular diseases in the future.

To the best of our knowledge, this is the first study to report an association of two *LIPC* SNPs, rs2043085 and rs1532085, with urinary 8-OHdG levels. The SNP rs2043085 has been associated with both HDL-C and fasting plasma glucose levels [44, 45] and is an expression quantitative trait locus (eQTL). By using Affymetrix Human array to analyze gene expression in 152 samples of liver tissues, Folkersen et al. [46] showed that the *LIPC* gene is the only gene located close to the SNP rs1532085, and its minor allele is associated with lower *LIPC* expression. The SNP rs1532085 is also an eQTL that interacts with other lipid metabolism genes to determine HDL-C levels and is a risk allele for Alzheimer disease and polypoidal choroidal vasculopathy [47–49]. Although these SNPs have a close relationship with lipids, our data demonstrated that their association with 8-OHdG levels were independent of HDL-C or TG levels because their association was not affected after the adjustment for HDL-C and TG levels. HL has been shown to affect the ROS by modulating the activity of PPARδ, a key transcription factor known to counteract ROS production [16–18] and urinary 8-OHdG is generated following the repair of ROS-mediated DNA damages [50]. Thus we suggested that the *LIPC* variants decreased *LIPC* transcription and, in turn HL activity which further increased the levels of urinary 8-OHdG, possibly due to the down regulation of HL in the transcription factor PPARδ. Intriguingly, rs1800588 was not linked to 8-OHdG levels, disagreeing with a previous finding of an association with levels of MDA-LDL, an oxidative stress marker [9]. This may be attributable to the limited size of our sample population, difference in ethnicity, heterogeneity of genome complexity, or a differential requirement of HL activity for lipid and nucleic acid oxidation. Additional studies are warranted to address the origin of this discrepancy and to clarify the causal link between HL activity and 8-OHdG production. In a PheWAS study conducted by Hall et al., significant associations were observed between rs1800588 and levels of folate and vitamin E, both of which were used as antioxidants to prevent atherosclerosis [12]. In addition, two *LIPC* SNPs were found to be associated with advanced age-related macular degeneration (AMD) [51–53], whose progression was characterized by a hallmark of age-related oxidative changes [54]. These data suggest that the *LIPC* locus is a key regulator of systemic oxidative stress.

We have previously demonstrated that sex and obesity interacted with two *LIPC* promoter polymorphisms to determine HDL-C levels in a Taiwanese population [8]. In this study, we further revealed that the association of the *LIPC* SNPs with HDL-C and 8-OHdG levels was predominantly observed in the men and those with high adiposity status. By contrast, their associations with serum TG levels were predominantly observed in the men and nonobese participants. Our findings are in line with those of previous studies, in which associations of multiple *LIPC* SNPs with HDL-C levels and risks of myocardial infarction and coronary artery disease were influenced by dietary intake, physical activity, sex, and obesity [10, 14, 47, 55–60]. These results suggest that the association of *LIPC* SNPs with various phenotypes should be individually assessed according to sex and obesity status.

In this study, we demonstrated that serum TG levels suppressed the relationship between *LIPC* SNPs and HDL-C levels. Several types of third variables calculated through statistical analyses, including mediation variables, confounding variables, suppression variables, and moderators [42], can be used to address the relationship between an independent and a dependent variable. Suppression variables increase the predictive validity of another variable by its inclusion into a regression equation. In general, the omission of a suppressor would underestimate the effect of an independent variable on a dependent variable, thereby reducing the magnitude of the relationship between the two variables. We have previously reported that this suppression effect may be biologically crucial, and it may partially explain the missing heritability in healthy individuals [38, 61, 62]. In brief, we previously demonstrated a suppression effect of sE-selectin levels on the association of *ABO* blood group genotypes with the TG-to-HDL-C ratio [38]. Furthermore, we demonstrated that adiponectin levels suppressed the association of *CDH13* genotypes with metabolic phenotypes, and the respective acute phase reaction protein levels reciprocally suppressed the association of *CRP/SAA* genotypes with SAA/CRP levels [61, 62]. The demonstration of the suppression effects of serum TG levels on the association of the *LIPC* SNPs with HDL-C levels in the men support the crucial role of the suppression effect in genetic association studies and further suggests that suppression is dependent on sex.

Pleiotropy observed in genetic association studies can provide insight into the shared biology underlying a spectrum of phenotypes. The existence of a real form of pleiotropy may be considered when associations with multiple phenotypes are observed on the level of an SNP, a gene, or a locus [63]. A recent study of the National Human Genome Research Institute’s catalogue of published genome-wide association studies reported that 4.6% of the SNPs and 16.9% of the genes have cross-phenotype effects [64]. However, these are believed to be underestimated because they rely on highly conservative criteria. Pleiotropy for *LIPC* SNPs has been widely reported. For instance, by resequencing the coding and the 5′ and 3′ untranslated regions of 78 candidate genes, Service et al. reported the association of *LIPC* SNPs with TC, HDL-C, and TG levels [65], which are the most commonly found associations for *LIPC* SNPs. In addition, different *LIPC* SNPs have been associated with levels of circulating MDA-LDL [9]; phospholipids and sphingolipids [66]; folate and vitamin E [12]; metabolic syndrome [44]; visceral adiposity indicators (VAI) and triglyceride and fasting plasma glucose (TyG) index-related parameters [37]; and diseases such as advanced AMD [52, 53, 67], coronary artery disease [10], and myocardial infarction [55]. Our finding of an association of the *LIPC* SNPs with urinary 8-OHdG levels contributes to the growing list of these pleiotropic associations and indicates that systemic oxidative stress is a likely underlying cause of HL-associated diseases. Therefore, future in-depth research on the outcomes of this oxidative stress is essential to prevent cardiovascular disease progression in high-risk populations.

## Limitations

The main limitation of this study is its moderate sample size with a relatively low number of subjects genotyped. Nevertheless, replication in a second cohort can improve the strength of the study, and the results regarding the association with HDL-C levels and interaction with sex and obesity were similar to those reported in our previous study. Another limitation is that we analyzed only three *LIPC* SNPs, which were incomplete and did not represent all the genetic variations of *LIPC*. In addition, multiple testing imposed another limitation on our study. Nevertheless, the statistical significance of a portion of our results with adjustment for the three studied SNPs became marginal when the Bonferroni correction was applied stringently to multiple tests.

## Conclusion

Our study results revealed the complexity of the association of variation of the *LIPC* locus with lipid profiles and oxidative stress, and the crucial effects of interaction and mediation on such associations. The association of the *LIPC* SNPs with urinary 8-OHdG levels may contribute to our understanding of the link between various *LIPC* SNPs and oxidative stress-related diseases such as advanced AMD. Thus, our findings can possibly provide a way to identify high-risk populations of cardiovascular diseases for preventive medicine. Subsequent effects on treatment procedures and prognoses are expected to be derived from this study.

## Additional file

Additional file 1: **Table S1.** Basic characteristics and genotyping assays for the *LIPC* gene variants. **Table S2.** Associations between the *LIPC* rs2043085 genotypes and metabolic traits and inflammatory marker levels. **Table S3.** Associations between the *LIPC* rs1532085 genotypes and metabolic traits and inflammatory marker levels. **Table S4.** Associations between the *LIPC* rs1800588 genotypes and metabolic traits and inflammatory marker levels. Additional file References. (DOC 118 kb)

## Abbreviations

8-OHdG: 8-hydroxy deoxyguanosine.
BMI: Body mass index
CRP: C-reactive protein
HDL-C: High-density lipoprotein cholesterol
HOMA-IR: Homeostasis model assessment of insulin resistance
LDL-C: Low-density lipoprotein cholesterol
MCP-1: Monocyte chemotactic protein-1
MMP1: Matrix metalloproteinase 1
MMP2: Matrix metalloproteinase 2
MMP9: Matrix metalloproteinase 9
SAA: Serum amyloid A
sE-selectin: soluble E-selectin
sICAM1: soluble intercellular adhesive molecule 1
SNPs: Single nucleotide polymorphisms
sP-selectin: soluble P-selectin
sTNFRII: soluble tumor necrosis factor-alpha receptor 2
sVCAM1: soluble vascular cell adhesive molecule 1
TG: Triglycerides
