# Supplementary material for: Pleiotropic association of LIPC variants with lipid and urinary 8-hydroxy deoxyguanosine levels in a Taiwanese population
Source: Lipids Health Dis. 2019 May 10;18:111. doi: 10.1186/s12944-019-1057-9 (PMC6511151; doi:10.1186/s12944-019-1057-9)
Supplement: Supplementary file 1 — Table S1. Basic characteristics and genotyping assays for the LIPC gene variants. Table S2. Associations between the LIPC rs2043085 genotypes and metabolic traits and inflammatory marker levels. Table S3. Associations between the LIPC rs1532085 genotypes and metabolic traits and inflammatory marker levels. Table S4. Associations between the LIPC rs1800588 genotypes and metabolic traits and inflammatory marker levels. Additional file References. (DOC 118 kb) [file 12944_2019_1057_MOESM1_ESM.doc]

Addotioanal file 1: Table S1. Basic characteristics and genotyping assays for the *LIPC* gene variants

| SNP number | Position | Location | Minor allele | MAF | HWE | Genotyping Assays |
| --- | --- | --- | --- | --- | --- | --- |
| rs2043085 | 58388755 | near gene 5’ | T | 0.464 | 0.967 | TaqMan SNP Genotyping Assays |
| rs1532085 | 58391167 | near gene 5’ | A | 0.455 | 0.898 | TaqMan SNP Genotyping Assays |
| rs1800588 | 58431476 | promoter | T | 0.367 | 0.943 | TaqMan SNP Genotyping Assays |

SNP: single nucleotide polymorphism; MAF：minor allele frequency; HWE：Hardy-Weinberg equilibrium

Addotioanal file 1: Table S2. Associations between the *LIPC* rs2043085 genotypes and metabolic traits and inflammatory marker levels.

| Genotypes (N) | 11 (159) | 12 (284) | 22 (119) | *P* | *P^＊^* |
| --- | --- | --- | --- | --- | --- |
| BMI | 23.6 ± 3.2 | 24.4 ± 3.5 | 24.5 ± 3.2 | 0.032 | 0.132 |
| Waist circumference (cm) | 83.2 ± 9.0 | 85.1 ± 9.6 | 86.6 ± 9.3 | 0.072 | 0.168 |
| Fasting plasma glucose (mg/dL) | 96.8 ± 25.0 | 96.6 ± 25.0 | 94.9 ± 11.1 | 0.385 | 0.166 |
| Fasting serum insulin (μU/mL) | 9.1 ± 5.2 | 9.0 ± 4.0 | 9.2 ± 4.6 | 0.337 | 0.141 |
| HOMA-IR | 2.2 ± 1.3 | 2.2 ± 1.3 | 2.3 ± 1.2 | 0.233 | 0.062 |
| Fibrinogen (mg/dL) | 257.8 ± 67.1 | 267.3 ± 68.1 | 247.8 ± 63.3 | 0.337 | 0.359 |
| CRP (mg/L) | 1.0 ± 1.4 | 1.0 ± 1.2 | 1.2 ± 1.5 | 0.127 | 0.293 |
| sE-selectin (ng/mL) | 50.0 ± 19.9 | 53.7 ± 26.1 | 54.1 ± 28.9 | 0.946 | 0.575 |
| sP-selectin (ng/mL) | 146.3 ± 119.0 | 140.5 ± 115.4 | 128.7 ± 103.4 | 0.132 | 0.078 |
| sVCAM1 (ng/mL) | 491.1 ± 105.0 | 488.8 ± 115.6 | 491.0 ± 195.3 | 0.821 | 0.928 |
| sICAM1 (ng/mL) | 238.7 ± 113.3 | 243.2 ± 107.9 | 236.0 ± 118.5 | 0.886 | 0.638 |
| TNFR2(pg/mL) | 3236.8 ± 938.4 | 3242.0 ± 984.2 | 3322.0 ± 750.7 | 0.357 | 0.443 |
| MCP-1(pg/mL) | 78.0 ± 66.1 | 74.6 ± 61.7 | 69.0 ± 45.4 | 0.354 | 0.291 |
| MMP1 (pg/mL) | 491.4 ± 1074.0 | 418.1 ± 953.0 | 581.6 ± 1658.0 | 0.947 | 0.965 |
| MMP2(ng/mL) | 127.8 ± 40.8 | 125.7 ± 42.5 | 129.2 ± 37.9 | 0.398 | 0.276 |
| MMP9 (ng/mL) | 132.6 ± 123.9 | 152.0 ± 117.0 | 129.5 ± 82.2 | 0.778 | 0.945 |
| SAA ( g /mL) | 5.4 ± 12.5 | 5.9 ± 12.8 | 4.2 ± 2.8 | 0.623 | 0.249 |

*P* adjusted for age, sex, BMI and current smoker; *P^＊^* adjusted for age, sex, BMI and current smoker and TG;

Addotioanal file 1: Table S3. Associations between the *LIPC* rs1532085 genotypes and metabolic traits and inflammatory marker levels.

| Genotypes (N) | 11 (114) | 12 (287) | 22 (162) | *P* | *P^＊^* |
| --- | --- | --- | --- | --- | --- |
| BMI | 24.5 ± 3.2 | 24.4 ± 3.5 | 23.6 ± 3.2 | 0.038 | 0.155 |
| Waist circumference (cm) | 86.3 ± 9.3 | 85.3 ± 9.6 | 83.2 ± 9.0 | 0.101 | 0.227 |
| Fasting plasma glucose (mg/dL) | 94.8 ± 11.1 | 96.7 ± 24.9 | 96.5 ± 24.8 | 0.495 | 0.228 |
| Fasting serum insulin (μU/mL) | 9.1 ± 4.3 | 9.1 ± 4.1 | 9.1 ± 5.2 | 0.295 | 0.116 |
| HOMA-IR | 2.1 ± 1.1 | 2.2 ± 1.3 | 2.2 ± 1.3 | 0.229 | 0.068 |
| Fibrinogen (mg/dL) | 248.8 ± 64.3 | 267.0 ± 67.6 | 256.9 ± 67.2 | 0.503 | 0.532 |
| CRP (mg/L) | 1.2 ± 1.5 | 1.0 ± 1.2 | 1.0 ± 1.4 | 0.172 | 0.379 |
| sE-selectin (ng/mL) | 53.8 ± 28.9 | 53.8 ± 26.3 | 50.1 ± 19.8 | 0.870 | 0.505 |
| sP-selectin (ng/mL) | 127.4 ± 101.5 | 140.4 ± 116.2 | 146.7 ± 117.8 | 0.093 | 0.052 |
| sVCAM1 (ng/mL) | 488.4 ± 198.4 | 489.9 ± 115.6 | 490.9 ± 104.3 | 0.668 | 0.912 |
| sICAM1 (ng/mL) | 237.6 ± 120.8 | 241.1 ± 106.7 | 240.9 ± 113.6 | 0.778 | 0.538 |
| TNFR2(pg/mL) | 3304.9 ± 755.1 | 3242.0 ± 979.5 | 3243.5 ± 942.2 | 0.496 | 0.604 |
| MCP-1(pg/mL) | 70.8 ± 46.0 | 73.8 ± 61.6 | 77.4 ± 65.6 | 0.560 | 0.470 |
| MMP1 (pg/mL) | 599.0 ± 1692.0 | 411.1 ± 948.3 | 493.2 ± 1064.2 | 0.975 | 0.955 |
| MMP2(ng/mL) | 128.7 ± 38.5 | 126.2 ± 42.3 | 127.5 ± 40.6 | 0.461 | 0.324 |
| MMP9 (ng/mL) | 128.5 ± 81.6 | 151.8 ± 116.8 | 132.6 ± 123.3 | 0.754 | 0.925 |
| SAA ( g /mL) | 4.3 ± 2.8 | 5.4 ± 10.9 | 6.1 ± 15.3 | 0.642 | 0.769 |

*P* adjusted for age, sex, BMI and current smoker; *P^＊^* adjusted for age, sex, BMI and current smoker and TG;

Addotioanal file 1: Table S4. Associations between the *LIPC* rs1800588 genotypes and metabolic traits and inflammatory marker levels.

| Genotypes (N) | 11 (228) | 12 (261) | 22 (74) | *P* | *P^＊^* |
| --- | --- | --- | --- | --- | --- |
| BMI | 24.4 ± 3.4 | 24.3 ± 3.4 | 23.5 ± 3.2 | 0.109 | 0.031 |
| Waist circumference (cm) | 85.4 ± 9.7 | 84.9 ± 9.1 | 82.9 ± 9.7 | 0.951 | 0.614 |
| Fasting plasma glucose (mg/dL) | 95.3 ± 19.2 | 96.6 ± 20.7 | 98.2 ± 36.1 | 0.176 | 0.368 |
| Fasting serum insulin (μU/mL) | 9.1 ± 4.9 | 9.0 ± 4.3 | 9.1 ± 4.1 | 0.454 | 0.667 |
| HOMA-IR | 2.2 ± 1.4 | 2.2 ± 1.1 | 2.2 ± 1.5 | 0.315 | 0.559 |
| Fibrinogen (mg/dL) | 267.4 ± 70.3 | 256.4 ± 64.9 | 252.9 ± 363.8 | 0.161 | 0.173 |
| CRP (mg/L) | 1.0 ± 1.3 | 1.1 ± 1.3 | 1.0 ± 1.6 | 0.825 | 0.789 |
| sE-selectin (ng/mL) | 53.3 ± 25.5 | 53.5 ± 26.0 | 48.7 ± 20.9 | 0.856 | 0.505 |
| sP-selectin (ng/mL) | 136.7 ± 98.5 | 141.8 ± 121.3 | 140.6 ± 131.1 | 0.657 | 0.499 |
| sVCAM1 (ng/mL) | 501.4 ± 166.0 | 485.7 ± 106.1 | 469.1 ± 103.6 | 0.065 | 0.117 |
| sICAM1 (ng/mL) | 247.3 ± 126.5 | 239.4 ± 91.9 | 221.9 ± 123.4 | 0.133 | 0.067 |
| TNFR2(pg/mL) | 3263.5 ± 962.8 | 3261.7 ± 929.4 | 3206.9 ± 801.5 | 0.738 | 0.857 |
| MCP-1(pg/mL) | 75.4 ± 56.0 | 73.0 ± 64.9 | 74.9 ± 54.3 | 0.953 | 0.853 |
| MMP1 (pg/mL) | 432.2 ± 1069.1 | 507.7 ± 1290.1 | 474.6 ± 1003.0 | 0.787 | 0.804 |
| MMP2(ng/mL) | 128.1 ± 44.6 | 128.2 ± 39.6 | 120.2 ± 33.7 | 0.244 | 0.342 |
| MMP9 (ng/mL) | 144.2 ± 102.5 | 135.8 ± 108.1 | 152.9 ± 152.8 | 0.628 | 0.475 |
| SAA ( g /mL) | 6.1 ± 13.9 | 4.8 ± 9.1 | 5.2 ± 9.7 | 0.627 | 0.514 |

*P* adjusted for age, sex, BMI and current smoker; *P^＊^* adjusted for age, sex, BMI and current smoker and TG;

**additional file 1: References:**

1. Chang PY, Wu TL, Tsao KC, et al. Microplate ELISAs for soluble VCAM-1 and ICAM-1. Ann Clin Lab Sci 2005; 35:312-7.

2. Tsao KC, Chang PY, Li CC, Wu TL, Sun CF, Wu JT. Development of a microplate ELISA for circulating E-selectin: assay characterization, comparison with a commercial kit, wand establishment of normal reference values. J Clin Lab Anal 2003; 17:97-101.

3. Wu TL, I Chen Tsai, Chang PY, et al. Establishment of an in-house ELISA and the reference range for serum amyloid A (SAA): complementarity between SAA and C-reactive protein as markers of inflammation. Clin Chim Acta 2007; 376:72-6.

4. Wu TL, Tsao KC, Chang CP, Li CN, Sun CF, Wu JT. Development of ELISA on microplate for serum C-reactive protein and establishment of age-dependent normal reference range. Clin Chim Acta 2002; 322:163-8.

5. Wu TL, Chang PY, Li CC, et al. Microplate ELISA for urine microalbumin: reference values and results in patients with type2 diabetes and cardiovascular disease. Ann Clin Lab Sci 2005; 35:149-54.

6. Chiou CC, Chang PY, Chan EC, et al. Urinary 8-hydroxydeoxyguanosine nd its analogs as DNA marker of oxidative stress: development of an ELISA and measurement in both bladder and prostate cancers. Clin Chim Acta 2003; 334:87-94.
